# A novel multiplex qPCR‑HRM assay for the simultaneous detection of four abortive zoonotic agents in cattle, sheep, and goats

**DOI:** 10.1038/s41598-023-39447-1

**Published:** 2023-07-28

**Authors:** Boitumelo M. Modise, Sununguko W. Mpoloka, Tirumala B. K. Settypalli, Joseph Hyera, Alda Natale, Letizia Ceglie, Nomakorinte Gcebe, Chandapiwa Marobela-Raborokgwe, Gerrit J. Viljoen, Giovanni Cattoli, Charles E. Lamien

**Affiliations:** 1 Botswana National Veterinary Laboratory, Private Bag 0035, Gaborone, Botswana; 2 Department of Biological Sciences, University of Botswana, Private Bag 00704, Gaborone, Botswana; 3 Animal Production and Health Laboratory, Joint FAO/IAEA Division of Nuclear Techniques in Food and Agriculture, Department of Nuclear Sciences and Applications, International Atomic Energy Agency, Wagramer Strasse 5, P.O. Box 100, 1400 Vienna, Austria; 4 Botswana Vaccine Institute, Private Bag 0031, Gaborone, Botswana; 5 Istituto Zooprofilattico Sperimentale delle Venezie (IZSVe), Viale dell’Università, 10, 35020 Legnaro, Italy; 6 Agricultural Research Council–Bacteriology and Zoonotic Diseases Diagnostic Laboratory, Onderstepoort Veterinary Research, Pretoria, South Africa

**Keywords:** Microbiology, Molecular biology

## Abstract

Abortifacient pathogens induce substantial economic losses in the livestock industry worldwide, and many of these pathogens are zoonotic, impacting human health. As *Brucella* spp., *Coxiella burnetii*, *Leptospira* spp., and *Listeria monocytogenes* cause abortion, rapid differential molecular diagnostic tests are needed to facilitate early and accurate detection of abortion to establish effective control measures. However, the available molecular methods are laborious, time-consuming, or costly. Therefore, we developed and validated a novel multiplex real-time polymerase chain reaction (qPCR) method based on high-resolution melting (HRM) curve analysis to simultaneously detect and differentiate four zoonotic abortifacient agents in cattle, goats, and sheep. Our HRM assay generated four well-separated melting peaks allowing the differentiation between the four zoonotic abortifacients. Out of 216 DNA samples tested, *Brucella* spp. was detected in 45 samples, *Coxiella burnetii* in 57 samples, *Leptospira* spp. in 12 samples, and *Listeria monocytogene*s in 19 samples, co-infection with *Brucella* spp*.* and *Coxiella burnetii* in 41 samples, and 42 samples were negative. This assay demonstrated good analytical sensitivity, specificity, and reproducibility. This is a valuable rapid, cost-saving, and reliable diagnostic tool for detecting individual and co-infections for zoonotic abortifacient agents in ruminants.

## Introduction

Abortion is a livestock production and health problem, causing significant economic losses worldwide. It would be of great importance to public health if prompted by microorganisms that cause human diseases^1,2^. Livestock production losses associated with abortion have marked negative impacts on the poorest communities who depend on livestock for their livelihood, food security, health, and social well-being^3,4^.

Causes of abortion are multifactorial and range from infectious to non-infectious factors. Infectious factors, including bacteria, fungi, protozoan, and viruses, contribute to up to 90% of abortions^5^. Among the infectious agents are bacteria such as *Brucella* spp., *Coxiella burnetii*, *Leptospira* spp., and *Listeria monocytogenes*, which play a significant role in inducing abortion in ruminants and also are zoonotic. Non-infectious causes include nutritional, genetic/chromosomal/metabolic, physical, stress, housing conditions, and transport^6–8^.

Brucellosis is caused by the bacteria of the genus *Brucella*, among which the zoonotic *Brucella abortus* and *Brucella melitensis* infect cattle and small ruminants, respectively^9,10^. *Coxiella burnetii,* a zoonotic pathogen classified in the genus *Coxiella* causes Query fever (Q fever)^11,12^ and affects many animal species, including cattle, sheep and goats. Members of the genus *Listeria* cause Listeriosis^13,14^. *Listeria monocytogenes* is pathogenic to several animal species, while *Listeria ivanovi*i is only pathogenic to ruminants, mainly sheep^15,16^. Members of the genus *Leptospira* cause leptospirosis*,* with zoonotic species *Leptospira interrogans* and *Leptospira borgpetersenii* primarily responsible for infection in ruminants^17,18^.

Identifying livestock abortion causes is difficult and costly worldwide, with success rates below 50% in abortion cases submitted to diagnostic laboratories^8,19,20^. In addition, the diversity of the causes of abortion makes diagnosis complex. Because abortions do not present specific premonitory signs in affected species, the identification of the aetiological agents can be challenging and frustrating. Diagnostics can be further complicated when multiple infectious agents co-exist in the same herd or episode, particularly in epidemic outbreaks^21^. Infectious agents represent the leading etiology and require effective disease control strategies that utilize rapid diagnosis to maintain healthy livestock and public health safety. Therefore, rapid differential diagnosis is critical and essential to detect and identify the zoonotic abortifacients causes of *Brucella* spp., *C. burnetii*, *Leptospira* spp*.*, and *L. monocytogenes.*

A multiplex PCR approach provides a good option for differential detection of zoonotic abortifacient pathogens. It has the advantage of being rapid and requires less input material and higher throughput when compared to monoplex PCR assays. Even though the multiplex qPCR assays using probes have proven to be highly effective^22–24^, their implementation is hindered by the expense associated with the probe. In addition, the probe is prone to degradation. An alternative to probe-based assays is an easy-to-perform, rapid, cost-effective diagnostic tool based on high-resolution melting (HRM) technology. HRM involves amplifying the target gene of interest by PCR in the presence of a fluorescent dye and subsequent melting of the amplicons by gradually increasing the temperature. The melting point (Tm) of double-stranded DNA is determined by monitoring the decline of fluorescence from a DNA intercalating dye bound to the double-stranded DNA as it denatures into single-stranded DNA at high temperatures^25^.

Currently, the HRM technology is used in different applications such as species detection and identification of clinical samples, differentiation of various pathogenic organisms, strain typing, genotyping, etc.^26–28^. However, no multiplex HRM real-time PCR method is available to simultaneously detect the pathogens covered in this study. Hence, we developed a multiplex HRM real-time PCR to simultaneously detect four zoonotic bacterial pathogens within a single reaction tube, *Brucella* spp., *Leptospira* spp. (targeting pathogenic species), *L. monocytogenes*, and *C. burnetii*, causing abortions in domestic ruminants.

## Results

### Assay design and optimization

The in-silico simulation with uMelt software showed that the predicted PCR amplicons for *Brucella* spp., *C. burnetii*, *L. monocytogenes* and *Leptospira* spp. had melting temperatures of 88.6, 85.5, 82.6 and 80.0 °C, respectively (Supplementary Fig. S1), suggesting that the expected PCR products could be suitable for differentiating these four bacterial pathogens by HRM. The analysis of the nucleotide content showed that *Brucella* spp. fragment had 101 nucleotides with a total GC content of 54.08%, followed by *C. burnetii* (47.05% GC content, 121 bp), *Leptospira* spp. (43.22% GC content, 77 bp) and *L. monocytogenes* (42.86% GC content, 93 bp).

Following the *in-silico* simulation, each primer set was evaluated in a monoplex reaction to optimize critical PCR parameters. Next, the four primers pairs were used in a multiplex assay, which was optimized to avoid primer-dimers and non-specific amplification.

**Figure 1:**
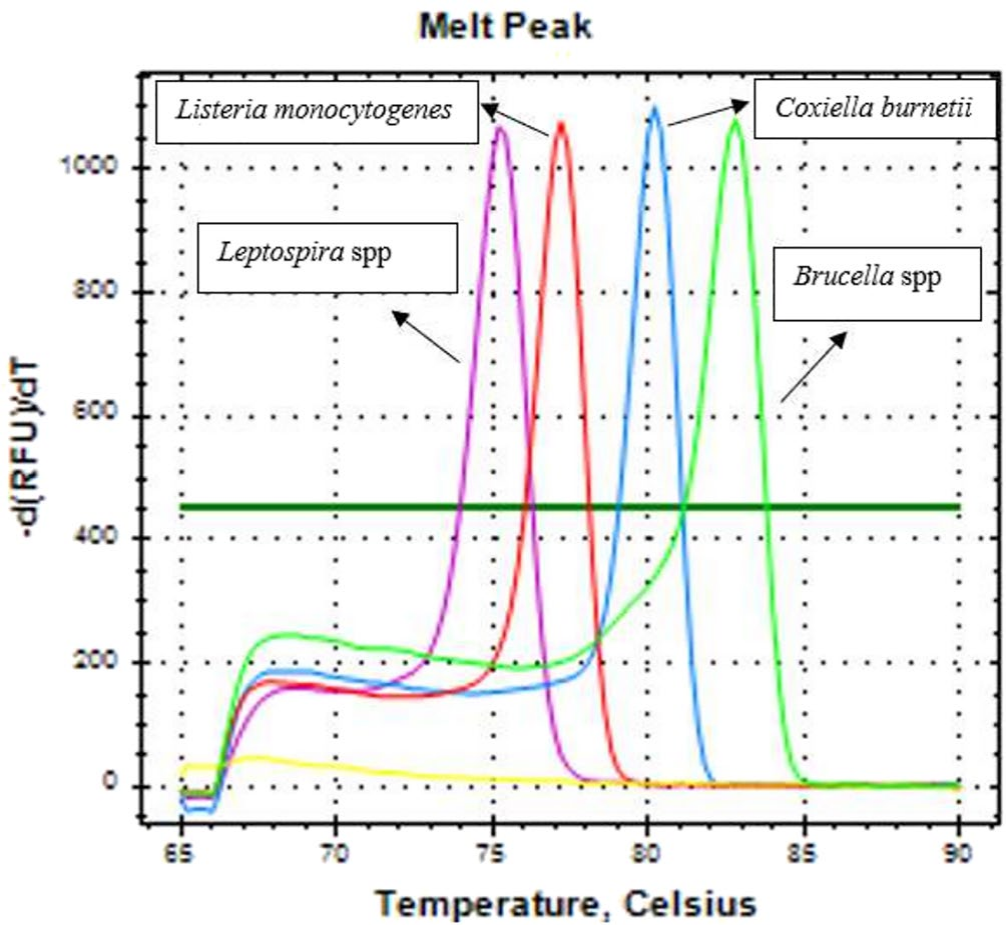
Preliminary evaluation results of multiplex qPCR-HRM assay using reference plasmids. The melting peaks of *Leptospira* spp. (pink), *L. monocytogenes* (red), *C. burnetii* (blue), and *Brucella* spp. (green). The melting curve and melting temperature are well separated.

**Figure 2:**
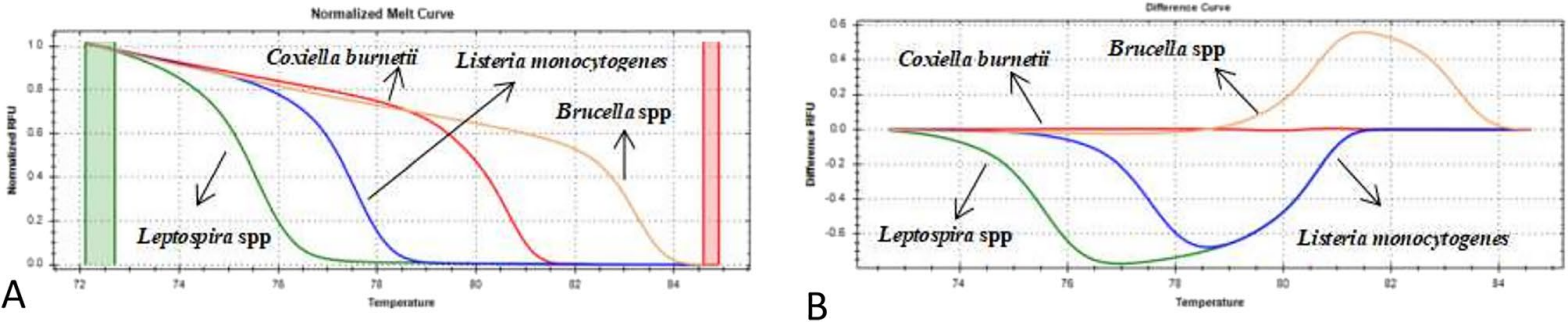
Normalized HRM plots of the PCR products of four zoonotic abortifacient bacteria. The normalized melt curve and difference curve plots are presented separately with different line colours for each pathogen: Normalized Melt Curve (**A**) and Difference Curve (**B**) of *Leptospira* spp. (green), *L. monocytogenes* (blue), *C. burnetii* (red), and *Brucella* spp. (orange). Green and red columns in the normalized melt curve plot represent pre-and post-melt normalization regions.

The preliminary evaluation of the multiplex qPCR-HRM assay using the positive control plasmids showed that the Tm values of the four pathogens were sufficiently separated: 83.2, 80.6, 77.4 and 75.6 °C for *Brucella* spp., *C. burnetii*, *L. monocytogenes* and *Leptospira* spp. respectively (Fig. 1) and expected amplicon size (Supplementary Fig. S2). In addition, the obtained normalized and difference plots also showed a clear differentiation of the four pathogens (Fig. 2).

### Limit of detection of the assay

The limits of detection with a 95% confidence of each pathogen were 10.20 (7.7–15.47), 4.26 (3.38–6.44), 6.72 (5.39–9.8), and 4.52 (3.62–6.75) copies per reaction for *Brucella* spp, *Leptospira* spp., *L. monocytogenes*, and *C*. *burnetii*, respectively.

### Repeatability and reproducibility (inter- and intra-assay variability)

The coefficients of variation between the runs were mainly in the range of 0.491 to 1.951, except for a higher concentration of *C. burnetii* (2.323), while the coefficients of variation within runs were from 0.089 to 1.493, except for a higher concentration in *C. burnetii* run 1 (3.339), and low (2.655) and medium (2.204) concentrations for *Leptospira* spp. run 1 (Table 1).

**Table 1:** Inter- and intra-assay variability of the multiplex qPCR-HRM assay.

Pathogen	Template concentration	Inter-assay variability (by Cq values)	Intra-assay variability (by Cq values)		
			Run 1	Run 2	Run 3
*Brucella* spp.	High (10^6^)	0.491	0.776	0.259	0.388
	Medium (10^4^)	0.536	0.530	0.089	0.103
	Low (10^2^)	1.741	0.585	0.264	0.214
*Listeria monocytogenes*	High (10^6^)	1.951	1.266	0.458	0.969
	Medium (10^4^)	0.858	0.737	0.382	0.483
	Low (10^2^)	0.535	0.384	0.494	0.444
*Coxiella burnetii*	High (10^6^)	2.323	3.339	0.287	0.208
	Medium (10^4^)	0.505	0.573	0.245	0.233
	Low (10^2^)	0.528	0.723	0.318	0.204
*Leptospira* spp.	High (10^6^)	1.512	1.064	1.358	1.493
	Medium (10^4^)	1.535	2.204	1.454	1.048
	Low (10^2^)	1.471	2.655	0.324	0.547

The variability was calculated based on the threshold values for the amplification of three different concentrations of controls; higher (10^6^), medium (10^4^), and lower concentrations (10^2^) of each targeted bacterial pathogen run at three different intervals.

### Cross-platform compatibility test (analysis of robustness)

**Figure 3:**
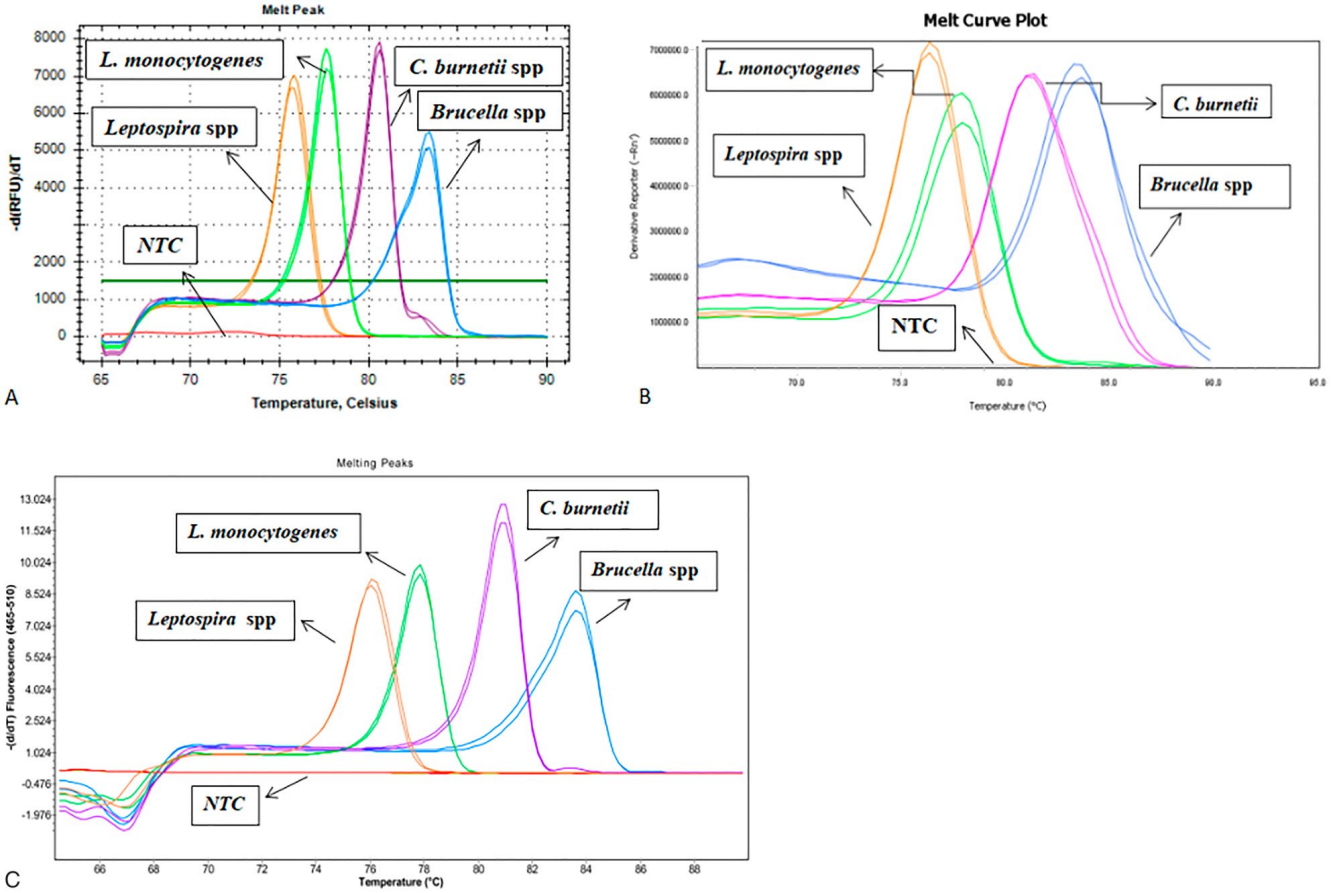
Melting peak analysis of the four zoonotic abortifacient bacteria; *Leptospira* spp (orange), *L. monocytogenes* (green), *C. burnetii* (purple), and *Brucella* spp. (blue), using different PCR platforms. (**A**) CFX96, Bio-Rad; (**B**) QuantStudio 6, Life Technologies; (**C**) LC480II, Roche.

In the cross-platform comparison, each real-time PCR instrument displayed a unique melting range for each pathogen (Table 2 and Fig. 3). Nevertheless, there was a constant but slight shift in the Tm values of the amplicons and melting peak shape from one instrument to another, as indicated in Table 2 and Fig. 3.

**Table 2:** Cross-platform analysis of the multiplex qPCR-HRM assay.

Pathogen	Real-time PCR machines with Tm values		
	CFX 96 (BioRad)	LC480ll (Roche)	QS6 (Life technologies)
*Brucella* spp.	83 ± 0.6 (82.4–83.6)	83.33 ± 0.44(82.89–83.77)	83.48 ± 0.79 (82.69–84.27)
L. *monocytogenes*	77.4 ± 0.4 (77.0–77.8)	77.69 ± 0.45 (77.24–78.14)	77.98 ± 0.5 (77.48–78.48)
*C. burnetii*	80.4 ± 0.6 (79.8–81.0)	80.82 ± 0.46 (80.36–81.28)	80.97 ± 0.5 (80.47–81.47)
*Leptospira* spp.	75.5 ± 0.5 (75.0–76.0)	75.99 ± 0.48 (75.51–76.47)	76.24 ± 0.5 (75.74–76.74)

Different real-time PCR instruments were used for assay evaluation with their respective amplicon melting temperature values indicated for the targeted four bacterial pathogens.

### Specificity, discriminating power, and assay performance

**Figure 4:**
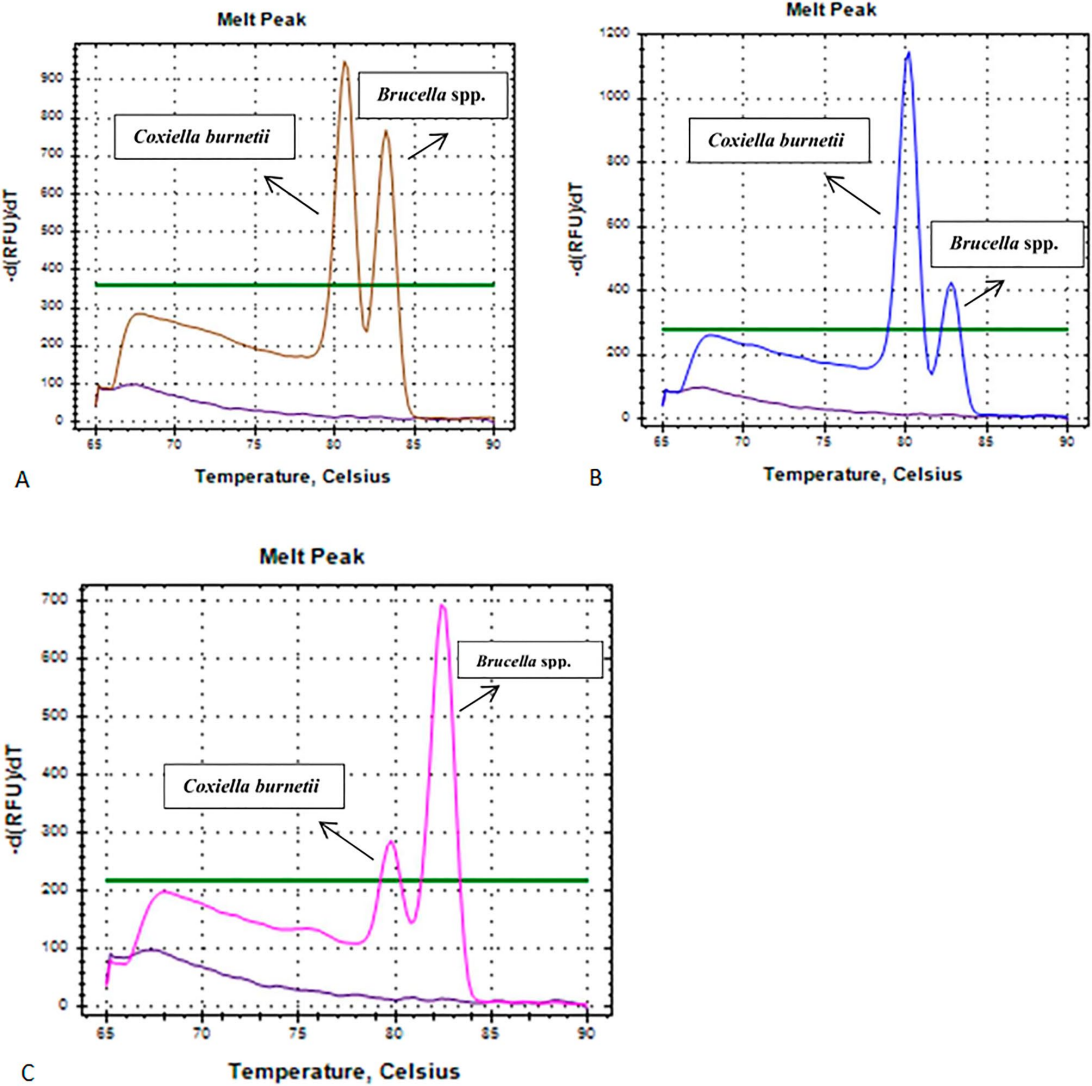
Melting peaks of samples presenting three different cases of co-infection with *Brucella* spp and *C. burnetii.*

**Figure 5:**
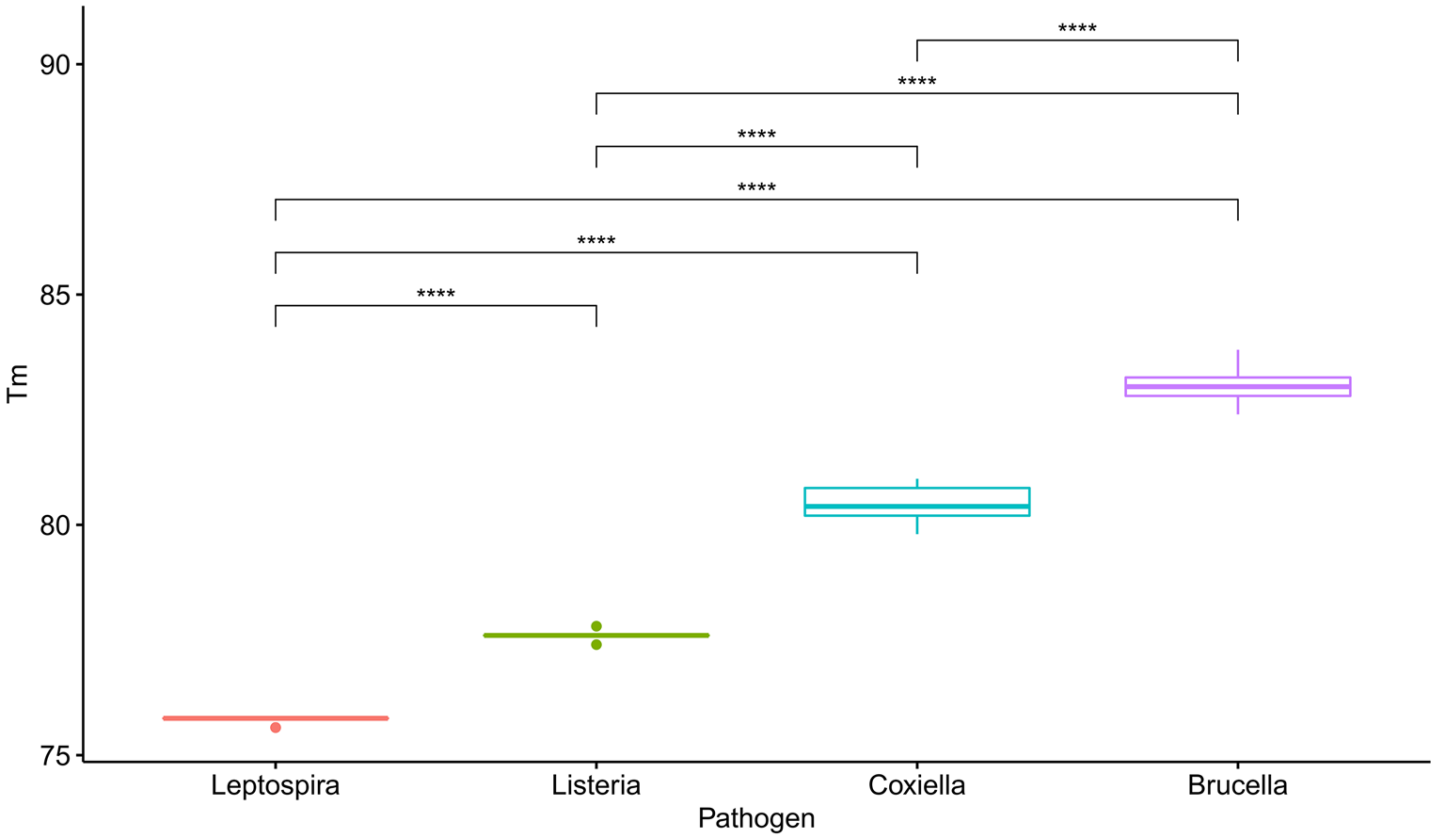
Box and whisker plot showing each pathogen's melting temperatures (Tm) in all tested samples. The differences in Tm for *Leptospira* spp., *Brucella* spp., *C. burnetii*, and *Listeria monocytogenes* are shown. Asterisks denote statistical significance (p ≤ 0.0001).

The assay's specificity, discriminating power, and performance were tested using 216 DNA from various samples (Supplementary Table S1). Of the 216 samples tested, *Brucella* spp. was detected in 45 samples, *C. burnetii* in 57 samples, *Leptospira* spp. in 12 samples, *L. monocytogenes* in 19 samples, and 42 samples were negative. Additionally, both *C. burnetii* and *Brucella* spp. were detected in 41 samples, indicating a co-infection with the two pathogens (Fig. 4). All the positive and negative results were further confirmed using respective previously established assays, and there was 100% agreement. Moreover, no cross-reactivity was observed, indicating that the multiplex assay accurately identified the four bacterial pathogens. The overall Tm ranges for each of the four pathogens are illustrated in Fig. 5 and show a clear separation between all the pathogens. The One-way ANOVA test showed that the average Tm was significantly different (p ≤ 0.0001) between each pair of the four pathogens (Fig. 5).

To further evaluate the specificity of the fourplex assay, non-target pathogen DNA samples (*E. coli*, *Pseudomonas aeruginosa*, *Staphylococcus aureus*, *Pasteurella multocida*, *Campylobacter* spp., *Trichomonas* spp., and *Salmonella* spp.) were tested, and no amplification was recorded.

## Discussion

This study reports the development and validation process of a multiplex qPCR-HRM assay for the simultaneous detection and differentiation of four bacterial pathogens known to cause abortions in domestic ruminants. The method specifically targeted important zoonotic abortifacients of cattle, sheep, and goats: *Leptospira* spp., *Brucella* spp., *C. burnetii*, and *L. monocytogenes*.

The multiplex qPCR-HRM assay successfully detected the four bacterial pathogens in a single run, thus making it suitable to screen abortive zoonotic diseases, saving time while accurately identifying the responsible pathogen. *Brucella* spp., *Leptospira* spp., *C. burnetii*, and *L. monocytogenes* were distinguished because of amplicon size and GC content differences that gave more effective discrimination between each pathogen through unique melting peaks. The HRM software further provided a better visual representation for the discrimination of the four bacterial pathogens by analyzing the melting of the PCR amplicons, mainly when several samples were analyzed in a single run.

The syndromic surveillance and multiplex testing approach used in this study enabled the diagnosis of abortion cases caused by pathogens not reported before in livestock in Botswana, such as Coxiella. Another great advantage shown in the study is the potential of our multiplex assay in detecting mixed infections. Co-infection with *C*. *burnetii* and *Brucella* spp. is rare but has been reported in the literature^29,30^. The simultaneous presence of * C. burnetii* and *Brucella* spp. in domestic ruminants implies concurrent exposure to both zoonotic diseases. Therefore, this assay could represent a good alternative for routine screening and extensive molecular epidemiological surveillance of cattle, sheep, and goats' abortions. Moreover, the developed multiplex assay demonstrated the “one health” value, by revealing the circulation of pathogens of great public health interest in the animal reservoir.

In the clinical samples from Botswana, we did not detect *Leptospira* spp. and *Listeria monocytogenes*. It may be due to the low incidence of these two pathogens compared to *Brucella* spp. and *C. burnetii*. Future studies are needed to pursue Leptospira and Listeria testing in Botswana using the developed assay actively.

With the sensitivity displayed by the current assay, it can be used as a laboratory diagnostic tool during syndromic abortion surveillance. In addition, the assay is highly specific as all the four targeted bacterial pathogens were identified accurately, with no inter-species cross-reactivity and no fluorescence signal detected in non-target pathogen DNA. Another advantage of the multiplex qPCR-HRM assay was its ability to be compatible with various qPCR platforms. Indeed, with all the three qPCR instruments tested in this study, the analysis of the melting curves was sufficient to differentiate and discriminate among all the targeted four bacterial pathogens. The only observed variation was a constant shift in Tm values across the platforms for each pathogen. Similarly, this kind of variation has previously been reported, possibly due to the variability in the fluorescence data collection mode and data analysis software^26^. Besides, being compatible with various qPCR platforms, the assay is easy to set up and interpret, becoming a potential tool for easy implementation in any veterinary and public health diagnostic laboratories, even with moderate resources.

Various techniques have been developed for identifying and detecting abortifacient agents affecting cattle, sheep, and goats^31–33^. However, these methods have many drawbacks, including being hazardous, arduous, laborious, and time-consuming^31–33^. An earlier study reported the development of a multiplex RT-PCR method to identify abortive agents in ruminants^23^. However, this assay is more expensive due to the need for seven different probes. In contrast, the present multiplex qPCR-HRM assay does not require any probe or labelled primers, making it a relatively cheap, user-friendly, and simple method to implement. Hence, by avoiding the probes, the present assay makes it more feasible to apply syndromic surveillance better to investigate abortions in ruminants.

The multiplex qPCR-HRM assay described here enables the simultaneous detection and differentiation of four bacteria causing abortions in ruminants: *Leptospira* spp., *Brucella* spp., *C. burnetii*, and *L. monocytogenes*. Therefore, this assay may save time in detecting abortifacient bacteria. The assay is easy to perform and interpret, cost-effective, reproducible, sensitive, and specific, thus providing a good opportunity for differential diagnosis, rapid screening, and syndromic surveillance of zoonotic abortifacient agents. The assay would significantly contribute to abortive disease management worldwide at the public health and veterinary level. However, further validation through inter-laboratory testing is called for before laboratories can implement the assay in routine diagnostics.

Further investigations are also required to genotype the leptospires responsible for abortion, correlate genotypes and serogroups, implement a vaccination plan, and hopefully target specific animal species able to act as a reservoir of this zoonotic infection. Genotyping of *C. burnetii* strains could also be useful to compare strains commonly isolated in cattle and caprine, and to correlate them to human isolates to understand their zoonotic potential.

## Materials and methods

### Ethical consideration

The present study followed international ethical guidelines and was evaluated and approved (UBR/RES/ACUC/016) by the Animal Care and Use Committee of the Office of Research and Development, University of Botswana. The Ministry of Agriculture approved permission to conduct the study and test samples from cattle, sheep and goats, with Reference No: MOA 1/15/4 II (6), and to publish the study, with Reference No: DVS/4/13/657029505 I (208). Samples used were provided by Botswana National Veterinary Laboratory (have been used for routine diagnosis). Authors confirm that this study is reported in accordance with ARRIVE guidelines (https://arriveguidelines.org).

### Samples and nucleic acid extraction

A total of 101 clinical samples from cattle, sheep, and goats collected in abortion cases originating from different geographical regions of Botswana were used for assay development (Supplementary Table S1). The 101 samples included DNAs, livers, spleens, ovary tubes, vaginal swabs, kidney bladder, lymph nodes, brains, placenta, abdominal/stomach contents, whole blood and sera stored at Botswana National Veterinary Laboratory (BNVL) from the period 2010 to 2021. In addition, 115 samples were included to validate the multiplex qPCR-HRM test method. These 115 samples comprised of 34 milk samples (from cattle) from BNVL Dairy Hygiene Unit used for routine quality control testing, four confirmed culture positive *L*. *monocytogenes* samples provided by University of Natural Resources and Life Sciences, Vienna, Austria, four confirmed *Brucella* spp. DNA from BNVL, 15 confirmed DNA samples (11 *Leptospira* spp. belonging to 11 different serovars and 8 serogroups, three *C. burnetii* detected in bovine and caprine abortions and milk and one *L. monocytogenes* strains) from different countries provided by Istituto Zooprofilattico Sperimentale delle Venezie (IZSVe), Legnaro, Italy, four confirmed *Brucella* spp. DNA provided by Veterinary Laboratories Agency (VLA)-Weybridge currently Animal and Plant Health Agency (APHA), UK and 54 confirmed DNA samples (24 *Brucella* spp. belonging to *Brucella abortus*, *Brucella melitensis*, *Brucella ovis* and *Brucella suis*, 15 *C. burnetii*, 14 *L. monocytogenes* and one *Leptospira* spp.) from Agricultural Research Council-Onderstepoort Veterinary Institute (ARC-OVI), Pretoria, South Africa.

Tissue suspensions (10% w/v) were prepared in sterile phosphate buffer saline (PBS), and swab samples were re-suspended in 0.5 ml PBS. Nucleic acid (DNA) was extracted from 200 μl of tissue suspensions, swab suspension, milk, sera, or blood using the DNeasy Blood & Tissue Kit (Qiagen, Hilden, Germany) as per the manufacturer's instructions with some modifications. The extracted DNA samples were stored at − 20 °C until further analysis.

### Positive controls

Synthetic plasmids harboring the target fragments were used as positive controls for *C. burnetii*, *Leptospira* spp., and *L. monocytogenes* except for *Brucella* spp. The plasmid for *C. burnetii* was sourced from Eurofins (GmbH, Germany), while for *Leptospira* spp. and *L. monocytogenes*, plasmids were manufactured by Eurogentec (Seraing, Belgium). For *Brucella* spp, BV9, NCTC 10507 *Brucella abortus* strain was used to produce positive control plasmids. Thus, the targeted gene was amplified by PCR using the primers designed (Table 3) for the multiplex qPCR-HRM assay. The PCR product (101 bp) was checked on 2% agarose gel, and amplicons were purified using Wizard® SV Gel and PCR Clean-Up System (Promega, Madison, USA) as per the manufacturer's instructions. The purified PCR products were cloned into pGEM®-T Vector system (Promega, Madison, WI, USA). The plasmids were sequenced commercially by LGC Genomics (Germany) to confirm the presence of the correct target. The concentrations of *C. burnetii*, *Leptospira* spp., *L. monocytogenes,* and *Brucella* spp. plasmids were determined fluorometrically using Quant-iT PicoGreen dsDNA Assay Kit (ThermoFisher Scientific, USA) and a NanoDrop 3300 Fluorospectrometer (Thermo Scientific, USA) and converted into copy numbers following the steps described previously^34^.

**Table 3:** List of the primers used in this study to amplify the targeted pathogens' fragments.

Pathogen	Target	Primer ID	Sequence (5ʹ → 3ʹ)	PCR product length (bp)	Total G+C content (%)
*Brucella* spp.	IS711	BruHRM_F	AAGCCGGATAGAAGGCTTGA	101	54.08
		BruHRM_R	CTGCATGCTGTTGTCGATG		
*C. burnetii*	IS1111	CoxHRM_F	AGGAGACACACCAACCGAGT	121	47.05
		CoxHRM_R	GGTTGATGCTTATCGGGCTA		
*Leptospira* spp.	LiPL32	LepHRM_F	CGGTTTAGTCGATGGAAACAA	77	43.22
		LepHRM_R	GAACTCCCATTTCAGCGATT		
*L. monocytogenes*	ssrA	LisHRM_F	CGGTAACAGGCTTCCATTCA	93	42.86
		LisHRM_R	GGGTCTCACTCTAAGTGGGCTA		

The names and sequences of the primers designed, target genes, and the estimated PCR amplicon size and G+C content are presented.

### Targeted genes and primer design

The existing monoplex real-time PCR tests for detection of *Brucella* spp., *C. burnetii*, *Leptospira*^22^ and *L. monocytogenes*^35^ were refined to design a multiplex real-time PCR assay using HRM technique for the detection of the four zoonotic pathogens. In brief, published gene sequences for *Brucella* spp. (IS711), *C. burnetii* (IS1111), *Leptospira* spp. (LiPL32), and *L. monocytogenes* (ssrA) were downloaded from GenBank and aligned using Clustal W in MEGA 7.0. Primers (Table 3) were designed on the conserved regions using the Primer 3 online tool (http://bioinfo.ut.ee/primer3-0.4.0/) to produce PCR amplicons of different size and different GC content to allow enough separation between the melting regions of the targeted pathogens under consideration. The specificity of each primer sequence was checked using the Basic Local Alignment Search Tool (NCBI/Primer-BLAST).

In-silico simulation using the uMelt software (https://www.dna.utah.edu/umelt/umelt.html) was performed to predict the melting temperatures of the expected PCR amplicons to avoid overlapping or similar Tm of the four pathogens. The total G+C content of the predicted PCR amplicons (Table 3) was calculated using BioEdit software package version 7.1.3.0. All primers were synthesized and purified by Eurofins, GmbH (Germany).

### Multiplex real-time PCR HRM (qPCR-HRM) assay development and optimization

The designed HRM primers were evaluated and optimized on a CFX96 Touch Real-Time PCR Detection System (Bio-Rad, USA) in monoplex reaction to select the most appropriate annealing temperature, and primer concentrations. Four plasmids harboring the target fragments of each pathogen were included as positive controls and ultrapure water as a negative control or no template control in each experiment. In the next step, the four primer pairs were pooled together to produce a multiplex assay, optimized like in a monoplex reaction, followed by the assay's standardization. The optimized conditions for the multiplex assay were as follows: a 20 μl PCR reaction volume containing 1 × SsoFast™ EvaGreen® Supermix (BioRad, Hercules, CA), 150 nM each primer pair of Brucella, Listeria and Coxiella, and 350 nM of Leptospira, and two μl DNA as a template. The PCR was carried out with initial denaturation at 95 °C for 5 min, 42 cycles with denaturation at 95 °C for 5 s, annealing at 65 °C for 4 s, and an extension at 70 °C for 5 s. Following the completion of PCR, products were subjected to the following melting program: denaturation at 95 °C for 1 min, cooling to 65 °C for 1 min, and continuous heating at 0.2 °C increments every 10 s with fluorescence acquisition from 65 to 90 °C.

High resolution Melting curve analysis was performed using the CFX Manager™ software version 3.1 (Bio-Rad) to analyze the amplification plots and melting graphs. The corresponding curves are displayed as negative first-derivative plots of fluorescence with respect to temperature. The data and melting profiles of the four bacteria were also analyzed using the Precision Melt Analysis™ Software version 1.2 (Bio-Rad). Normalized melt curves and difference plots were obtained by analyzing the active melt region and designating the corresponding pre-and-post melt regions.

### Limit of detection of the assay

The limit of detection of the multiplex assay was determined for each bacterial plasmid at dilutions of 20, 16, 12, 8, 4, 2, and 0 copies/reaction in pentaplicate on five separate days. The total proportions of positive results were recorded and subjected to Probit regression analysis using Ecotox package in R version 4.1.1. The detection limits were expressed as the lowest number of copies per reaction or lowest dilution with amplification of all the replicates, at a 95% confidence.

### Repeatability and reproducibility (Inter- and Intra-assay variability)

Intra- and inter-assay variability for the multiplex assay was determined at high, medium, and low copy numbers (10^6^, 10^4^, and 10^2^) using the quantification cycle (Cq) generated by amplification of diluted plasmids for the four pathogens. For intra-assay variability, the dilutions were run in five replicates in the same run, while for inter-assay variability, each dilution was tested in pentaplicates on three alternate days. Repeatability and reproducibility were estimated by computing the coefficients of variation between and within runs.

### Cross-platform compatibility test (analysis of robustness)

The cross-platform compatibility of this assay was evaluated on various real-time PCR instruments using the same PCR mix and protocol, relevant plasmids, and bacterial DNA extracted from clinical samples. The real-time PCR instruments used were CFX96 Touch Real-Time PCR Detection System (Bio-Rad Laboratories), LightCycler® 480 Real-Time PCR Systems (Roche), and QuantStudio™ 6 Flex Real-Time PCR System (Life Technologies).

### Specificity, discriminating power of the assay

The specificity and the discriminating power of the multiplex qPCR-HRM assay were demonstrated by testing nucleic acids from clinical samples from BNVL, culture samples provided by University of Natural Resources and Life Sciences, Vienna, Austria, and DNA samples provided by IZSVe, Italy, VLA (APHA), UK and ARC-OVI, SA. The status of DNA samples obtained from Italy, Austria, UK and SA and four samples from Botswana, was known. In contrast, the status of DNA samples from cattle, sheep, and goats collected in abortion cases from different geographical locations of Botswana was unknown. The details of the samples, i.e., sample source, sample type, and results using the multiplex real-time PCR assay, are summarized in the supplementary information (Table S1). All the samples were further confirmed by previously established real-time PCR methods^22,35–37^.

The assay's specificity was further evaluated by testing DNA extracted from cultured reference organisms from non-target, closely related bacterial pathogens, such as *E. coli* (ATCC® 25922™), *Pseudomonas aeruginosa* (ATCC® 25619™), *Staphylococcus aureus* (ATCC® 25923™), *Pasteurella multocida* (ATCC® 12945™), *Campylobacter* spp. (proficiency test sample) and *Salmonella* spp. (ATCC® 14028) and *Trichomonas* spp. (proficiency test sample).

One-Way ANOVA test and Tukey multiple comparisons of means were performed using R statistical software to determine whether the average Tm between the pathogens included in the multiplex panel were statistically different. Additionally, box and whisker plots were constructed to illustrate the differences between the Tm of the pathogens using the ggplot2 package in R.

## Supplementary Information

Supplementary Information.

## Data Availability

All data generated or analyzed during this study are included in the manuscript. Additional data is included in the Supplementary Information file.

## References

[1] van Engelen E, Luttikholt S, Peperkamp K, Vellema P, van den Brom R (2014). Small ruminant abortions in the Netherlands during lambing season 2012–2013. Vet. Rec..

[2] Vidal S (2017). Neglected zoonotic agents in cattle abortion: Tackling the difficult to grow bacteria. BMC Vet. Res..

[3] Abdelhadi FZ (2015). Abortions in cattle on the level of Tiaret Area (Algeria). Glob. Vet..

[4] Njiro SM (2011). A study of some infectious causes of reproductive disorders in cattle owned by resource-poor farmers in Gauteng Province, South Africa. J. S. Afr. Vet. Assoc..

[5] Parthiban S (2015). Review on emerging and reemerging microbial causes in bovine abortion. Int. J. Nutr. Food Sci..

[6] Beuzon CR (1997). Identification of *Salmonella **abortusovis* by PCR amplification of a serovar-specific IS200 element. Appl. Environ. Microbiol..

[7] Borel N (2014). Laboratory diagnosis of ruminant abortion in Europe. Vet. J..

[8] Clothier K, Anderson M (2016). Evaluation of bovine abortion cases and tissue suitability for identification of infectious agents in California diagnostic laboratory cases from 2007 to 2012. Theriogenology.

[9] Azam S (2016). Genetic characterization and comparative genome analysis of *Brucella **melitensis* isolates from India. Int. J. Genomics.

[10] Ducrotoy MJ (2015). Narrative overview of animal and human brucellosis in Morocco: Intensification of livestock production as a driver for emergence?. Infect. Dis. Poverty.

[11] Beare PA (2006). Genetic diversity of the Q fever agent, *Coxiella **burnetii*, assessed by microarray-based whole-genome comparisons. J. Bacteriol..

[12] Raoult D, Marrie T, Mege J (2005). Natural history and pathophysiology of Q fever. Lancet Infect. Dis..

[13] Hage E (2014). Identification of six Listeria species by real-time PCR assay. Lett. Appl. Microbiol..

[14] Weller D, Andrus A, Wiedmann M, den Bakker HC (2015). *Listeria **booriae* sp. nov. and *Listeria **newyorkensis* sp. nov., from food processing environments in the USA. Int. J. Syst. Evol. Microbiol..

[15] Hernandez-Milian A, Payeras-Cifre A (2014). What is new in Listeriosis?. Biomed. Res. Int..

[16] Vázquez-Boland JA, Domínguez-Bernal G, González-Zorn B, Kreft J, Goebel W (2001). Pathogenicity islands and virulence evolution in Listeria. Microb. Infect..

[17] Evangelista KV, Coburn J (2010). Leptospira as an emerging pathogen: A review of its biology, pathogenesis and host immune responses. Future Microbiol..

[18] Kumbhare MR, Surana AR, Arote RA, Borse GD (2019). Current status of leptospirosis: A zoonotic tropical disease. Int. J. Microbiol. Curr. Res..

[19] Wolf-Jäckel GA (2020). Diagnostic studies of abortion in Danish cattle 2015–2017. Acta Vet. Scand..

[20] Wolf-Jäckel GA (2021). Bovine abortions revisited—enhancing abortion diagnostics by 16S rDNA amplicon sequencing and fluorescence in situ hybridization. Front. Vet. Sci..

[21] Macías-Rioseco M (2019). Abortion outbreak caused by *Campylobacter **fetus* subspecies venerealis and *Neospora** caninum* in a bovine dairy herd. Rev. Mex Cienc. Pecu.

[22] Liu J (2016). Development of a TaqMan array card for acute-febrile-illness outbreak investigation and surveillance of emerging pathogens, including ebola virus. J. Clin. Microbiol..

[23] Sebastiani C (2018). A multi-screening Fast qPCR approach to the identification of abortive agents in ruminants. J. Microbiol. Methods.

[24] Selim AM, Elhaig MM, Gaede W (2014). Sviluppo di un test multiplex real-time PCR per il rilevamento di *Brucella* spp., *Leptospira* spp. e *Campylobacter foetus*. Vet. Ital..

[25] von Keyserling H, Bergmann T, Wiesel M, Kaufmann AM (2011). The use of melting curves as a novel approach for validation of real-time PCR instruments. Biotechniques.

[26] Gelaye E (2017). A novel HRM assay for the simultaneous detection and differentiation of eight poxviruses of medical and veterinary importance. Sci. Rep..

[27] Gopaul KK (2014). Development and assessment of multiplex high resolution melting assay as a tool for rapid single-tube identification of five *Brucella* species. Sci. Rep..

[28] Landolt P, Stephan R, Scherrer S (2019). Development of a new high resolution melting (HRM) assay for identification and differentiation of *Mycobacterium tuberculosis* complex samples. Sci. Rep..

[29] Song J (2021). *Rickettsia **burneti* and *Brucella **melitensis* co-infection: A case report and literature review. BMC Microbiol..

[30] Peric L (2018). Imported brucellosis and Q-fever coinfection in Croatia: A case report. J. Infect. Dev. Countries.

[31] Kahn LH (2006). Confronting zoonoses, linking human and veterinary medicine. Emerg. Infect. Dis..

[32] Maurin M, Raoult D (1999). Q fever. Clin. Microbiol. Rev..

[33] Limmathurotsakul D (2012). Fool’s gold: Why imperfect reference tests are undermining the evaluation of novel diagnostics: A reevaluation of 5 diagnostic tests for leptospirosis. Clin. Infect. Dis..

[34] Lamien CE (2011). Real time PCR method for simultaneous detection, quantitation and differentiation of capripoxviruses. J. Virol Methods.

[35] Jin D (2012). Rapid molecular identification of *Listeria* species by use of real-time PCR and high-resolution melting analysis. FEMS Microbiol. Lett..

[36] Barkallah M (2014). Survey of infectious etiologies of bovine abortion during mid- to late gestation in dairy herds. PLoS ONE.

[37] Loftis AD (2006). Surveillance of Egyptian fleas for agents of public health significance: Anaplasma, Bartonella, Coxiella, Ehrlichia, Rickettsia, and Yersinia pestis. Am. Soc. Trop. Med. Hygiene.

