# Supplementary material for: A novel multiplex qPCR‑HRM assay for the simultaneous detection of four abortive zoonotic agents in cattle, sheep, and goats
Source: Sci Rep. 2023 Jul 28;13:12282. doi: 10.1038/s41598-023-39447-1 (PMC10382562; doi:10.1038/s41598-023-39447-1)
Supplement: Supplementary file 1 — Supplementary Information. [file 41598_2023_39447_MOESM1_ESM.pdf]

A novel multiplex qPCR-HRM assay for the simultaneous detection of four abortive zoonotic agents in cattle, sheep, and goats

Boitumelo Magret Modise[1,2*], Sununguko Wata Mpoloka[2], Tirumala Bharani Kumar Settypalli[3], Joseph Hyera[4], Alda Natale[5], Letizia Ceglie[5], Nomakorinte Gcebe[6], Chandapiwa Marobela-Raborokgwe[1], Gerrit Johannes Viljoen[3], Giovanni Cattoli[3], Charles Euloge Lamien[3].

[1] Botswana National Veterinary Laboratory, Private Bag 0035, Gaborone, Botswana.

[2] University of Botswana, Department of Biological Sciences, Private Bag 00704, Gaborone, Botswana.

[3] Animal Production and Health Laboratory, Joint FAO/IAEA Division of Nuclear Techniques in Food and Agriculture, Department of Nuclear Sciences and Applications, International Atomic Energy Agency, Wagramer Strasse 5, P.O. Box 100, A-1400 Vienna, Austria.

[4] Botswana Vaccine Institute, Private Bag 0031, Gaborone, Botswana.

[5] Istituto Zooprofilattico Sperimentale delle Venezie (IZSVe), Viale dell'Università, 10 35020 Legnaro, Italy.

[6] Agricultural Research Council–Bacteriology and Zoonotic Diseases Diagnostic Laboratory, Onderstepoort Veterinary Research, Pretoria, South Africa

*Corresponding author email: boikensmod@gmail.com

Supplementary information

Supplementary Table S1: Samples tested by the multiplex qPCR-HRM assay for this study.

Identification	Country	District	DNA. source	Species	Tm	Pathogen	Status
Bot 1894/2014	Botswana	Kasane	SC	Bovine	82.8	Brucella	Blind
Bot 3354/2014	Botswana	Gaborone	Tissues	Caprine	80	Coxiella	Blind
					82.8	Brucella	
Bot 3065/2015	Botswana	Gaborone	Placenta	Ovine	80.2	Coxiella	Blind
					82.8	Brucella	
Bot 667/2015	Botswana	Nata	Tissues	Caprine	83.4	Brucella	Blind
Bot 2881/2016	Botswana	Pitsane	SC	Bovine	80.2	Coxiella	Blind
					83	Brucella	
Bot 1310/2017	Botswana	Nata	Tissues	Caprine	80.2	Coxiella	Blind
					82.6	Brucella	
Bot 1162/2017	Botswana	Mochudi	Tissues	Caprine	80.8	Coxiella	Blind
					83.4	Brucella	
Bot 1396/2017	Botswana	Gaborone	AF/contents	Ovine	80.8	Coxiella	Blind
Bot 1396/2017	Botswana	Gaborone	Tissues	Ovine	82.8	Brucella	Blind
Bot 2756/2017	Botswana	Ramotswa	SC	Caprine	80.4	Coxiella	Blind
					83	Brucella	
Bot 2756/2017	Botswana	Ramotswa	Tissues	Caprine	80.6	Coxiella	Blind
Bot 2928/2017	Botswana	Mahalapye	Tissues	Caprine	80.6	Coxiella	Blind
					83	Brucella	
Bot 2884/2017	Botswana	Ramotswa	Tissues	Caprine	80.6	Coxiella	Blind
					83.2	Brucella	
Bot 1309/2017	Botswana	Nata	Tissues	Caprine	80.2	Coxiella	Blind
Bot 3616/2017	Botswana	Ramotswa	Placenta	Bovine	80.4	Coxiella	Blind
					82.8	Brucella	
Bot 2930/2017	Botswana	Mahalapye	Tissues	Caprine	80.2	Coxiella	Blind
					82.8	Brucella	
Bot 4077/2017	Botswana	Molepolole	Tissues	Caprine	80.2	Coxiella	Blind
Bot 3907/2017	Botswana	Mahalapye	Tissues	Caprine	80.2	Coxiella	Blind
					82.6	Brucella	

Bot 1315/2017	Botswana	Nata	Tissues	Caprine	80	Coxiella	Blind
					82.8	Brucella	
Bot 893/2017	Botswana	Gaborone	Tissues	Caprine	80.6	Coxiella	Blind
Bot 4122/2017	Botswana	Letlhakane	Swab A	Caprine	81	Coxiella	Blind
					83.6	Brucella	
Bot 4122/2017	Botswana	Letlhakane	Swab B	Caprine	81	Coxiella	Blind
Bot 2757/2017	Botswana	Ramotswa	Tissues	Caprine	80.2	Coxiella	Blind
Bot 807/2017	Botswana	Ramotswa	Tissues	Caprine	80.2	Coxiella	Blind
Bot 1327/2018	Botswana	Mochudi	Tissues	Caprine	80.8	Coxiella	Blind
Bot 4326/2019	Botswana	Lobatse	Tissues	Bovine	80.4	Coxiella	Blind
					83	Brucella	
Bot 1528/2019	Botswana	Lobatse	Cultured tissue -DNA	Bovine	80.6	Coxiella	Blind
					83	Brucella	
Bot 1530/2019	Botswana	Lobatse	Cultured tissue -DNA	Bovine	80.6	Coxiella	Blind
					83.2	Brucella	
Bot 4258/2019	Botswana	Lobatse	Cultured tissue -DNA	Bovine	80.6	Coxiella	Blind
Bot 1676/2019	Botswana	Ghanzi	SC	Caprine	80.2	Coxiella	Blind
					82.8	Brucella	
Bot 1530/2019	Botswana	Lobatse	Tissues	Bovine	80.8	Coxiella	Blind
					83.4	Brucella	
Bot 3303/2019	Botswana	Lobatse	Tissues	Bovine	80	Coxiella	Blind
					82.4	Brucella	
Bot 1359/2019	Botswana	Mochudi	Tissues	Caprine	79.8	Coxiella	Blind
					82.4	Brucella	
Bot 2859/2020	Botswana	Lobatse	Serum	Bovine	80	Coxiella	Blind
					82.8	Brucella	
Bot 3090/2020	Botswana	Gaborone	Placenta	Ovine	80.2	Coxiella	Blind
Bot 2330/2020	Botswana	Ghanzi	Tissues	Caprine	80.4	Coxiella	Blind
Bot 2395/2020	Botswana	Molepolole	SC	Caprine	80.6	Coxiella	Blind
					83	Brucella	
Bot 1109/2020	Botswana	Gaborone	FT	Ovine	80.8	Coxiella	Blind
					83.2	Brucella	
Bot 1792/2020	Botswana	Mochudi	SC	Caprine	83	Brucella	Blind
Bot 1340/2020	Botswana	Ramotswa	Placenta	Ovine	80.4	Coxiella	Blind
					83	Brucella	

Bot 2833/2020	Botswana	Mochudi	FT	Caprine	80	Coxiella	Blind
Bot 2833/2020	Botswana	Mochudi	SC	Caprine	79.8	Coxiella	Blind
					82.4	Brucella	
Bot 2138/2020	Botswana	Mahalapye	Tissues	Caprine	79.8	Coxiella	Blind
Bot 1909/2020	Botswana	Lobatse	SC	Ovine	80	Coxiella	Blind
Bot 1571/2020	Botswana	Lobatse	Tissues A	Bovine	80	Coxiella	Blind
					82.6	Brucella	
Bot 1571/2020	Botswana	Lobatse	Serum	Bovine	82.6	Brucella	Blind
Bot 1571/2020	Botswana	Lobatse	Tissues B	Bovine	80.6	Coxiella	Blind
Bot 2933/2020	Botswana	Gaborone	Tissues	Caprine	80.2	Coxiella	Blind
					83	Brucella	
Bot 2132/2020	Botswana	Mochudi	Tissues	Caprine	80	Coxiella	Blind
					83	Brucella	
Bot 2112/2020	Botswana	Maun	Tissues	Caprine	80.4	Coxiella	Blind
Bot 3175/2020	Botswana	Shakawe	Tissues	Caprine	80.4	Coxiella	Blind
Bot 2046/2020	Botswana	Ghanzi	FT	Caprine	80.4	Coxiella	Blind
					83.2	Brucella	
Bot 2256/2020	Botswana	Ramotswa	Tissues	Caprine	80.2	Coxiella	Blind
Bot 2262/2020	Botswana	Mochudi	SC	Caprine	80.4	Coxiella	Blind
					82.6	Brucella	
Bot 2262/2020	Botswana	Mochudi	Tissues	Caprine	80	Coxiella	Blind
Bot 1920/2020	Botswana	Maun	SC	Bovine	80	Coxiella	Blind
					82.6	Brucella	
Bot 3677/2020	Botswana	Lobatse	Serum	Bovine	80	Coxiella	Blind
					82.6	Brucella	
Bot 2428/2020	Botswana	Molepolole	SC	Caprine	80	Coxiella	Blind
Bot 1883/2020	Botswana	Molepolole	SC	Caprine	79.9	Coxiella	Blind
Bot 2140/2020	Botswana	Mahalapye	Tissues	Caprine	80	Coxiella	Blind
Bot 686/2020	Botswana	Lobatse	Tissues	Bovine	83	Brucella	Blind
Bot 3098/2020	Botswana	Lobatse	Milk	Bovine	80.4	Coxiella	Blind
Bot 2535/2020	Botswana	Gaborone	Milk	Bovine	83.2	Brucella	Blind
Bot 3350/2020	Botswana	Lobatse	Milk	Bovine	82.6	Brucella	Blind
Bot 2799/2020	Botswana	Ghanzi	Milk A	Bovine	80.6	Coxiella	Blind
					82.8	Brucella	
Bot 2799/2020	Botswana	Ghanzi	Milk B	Bovine	80.8	Coxiella	Blind
Bot 1560/2020	Botswana	Lobatse	Milk	Bovine	83.2	Brucella	Blind
Bot 1509/2020	Botswana	Gaborone	Milk	Bovine	82.8	Brucella	Blind
Bot 2099/2020	Botswana	Lobatse	Milk	Bovine	80.4	Coxiella	Blind
Bot 1610/2020	Botswana	Lobatse	Milk	Bovine	80.2	Coxiella	Blind

Bot 2669/2020	Botswana	Lobatse	Milk	Bovine	80.4	Coxiella	Blind
Bot 2665/2020	Botswana	Lobatse	Milk	Bovine	80.4	Coxiella	Blind
Bot 1973/2020	Botswana	Gaborone	Milk	Bovine	80.8	Coxiella	Blind
					83	Brucella	
Bot 1611/2020	Botswana	Lobatse	Milk	Bovine	80.8	Coxiella	Blind
					83.2	Brucella	
Bot 2101/2020	Botswana	Serowe	Milk	Bovine	80.4	Coxiella	Blind
					82.8	Brucella	
Bot 1632/2020	Botswana	Gaborone	Milk	Bovine	80.2	Coxiella	Blind
Bot 1728/2020	Botswana	Gaborone	Milk	Bovine	80.4	Coxiella	Blind
Bot 2533/2020	Botswana	Gaborone	Milk	Bovine	80	Coxiella	Blind
Bot 808/2021	Botswana	Ramotswa	Swab	Caprine	80	Coxiella	Blind
					82.8	Brucella	
Bot 808/2021	Botswana	Ramotswa	WB	Caprine	80.6	Coxiella	Blind
Bot 808/2021	Botswana	Ramotswa	SC	Caprine	80	Coxiella	Blind
Bot 3345/2020	Botswana	Tsabong	Tissues	Caprine	80.2	Coxiella	Blind
Bot 1405/2021	Botswana	Ramotswa	SC	Caprine	80.6	Coxiella	Blind
Bot 523/2021	Botswana	Mahalapye	FT	Caprine	80.6	Coxiella	Blind
Bot 1368/2021	Botswana	Molepolole	SC	Caprine	80.8	Coxiella	Blind
					83.2	Brucella	
Bot 1048/2021	Botswana	Nata	SC	Caprine	80.6	Coxiella	Blind
Bot 1048/2021	Botswana	Nata	FT	Caprine	80.8	Coxiella	Blind
					83.2	Brucella	
Bot 952/2021	Botswana	Gaborone	SC	Caprine	80.8	Coxiella	Blind
					83.4	Brucella	
Bot 952/2021	Botswana	Gaborone	Tissues	Caprine	80.4	Coxiella	Blind
Bot 1175/2021	Botswana	Nata	SC	Caprine	80.2	Coxiella	Blind
					82.6	Brucella	
Bot 654/2021	Botswana	Nata	Serum	Bovine	82.8	Brucella	Blind
Bot 1064/2021	Botswana	Lobatse	FT	Bovine	83.4	Brucella	Blind
Bot 33/2021	Botswana	Lobatse	FT	Bovine	82.8	Brucella	Blind
ATCC 15313 GLi 11/2007	Austria	NK	NK	Rabbit	77.6	Listeria	known
ATCC 19114 GLi 18/2008	Austria	NK	Brain	Ruminant	77.6	Listeria	known
NCTC 10890 GLi 20/2008	Austria	NK	Faeces	Human	77.6	Listeria	known
GLI 22/2008	Austria	NK	NK	Poultry	77.6	Listeria	known
2776-2018	Italy	NK	Cheese	NA	77.8	Listeria	known

57448 C9-2018	Italy	NK	Foetus	Bovine	80.6	Coxiella	known
57448 C11-2018	Italy	NK	Foetus	Bovine	80.8	Coxiella	known
Milk Bolzano-reference material	Italy	Bozen	Milk	Caprine	80.8	Coxiella	known
Leptospira _ Icterohaemorrhagiae_ Bianchi	Italy	Pavia	Strain in EMJH medium	Man	75.8	Leptospira	known
Leptospira _ Canicola _ Alarik	Netherlands	NK	Strain in EMJH medium	Dog	75.8	Leptospira	known
Leptospira _ Grippothyphosa _Moskva V	Russia	Rostov	Strain in EMJH medium	Man	75.8	Leptospira	known
Leptospira _ Copenhageni _Wijmberg	Netherlands	NK	Strain in EMJH medium	Man	75.8	Leptospira	known
Leptospira _ Pomona _ Pomona	Australia	North Queensland	Strain in EMJH medium	Man	75.8	Leptospira	known
Leptospira _ Bratislava _Riccio 2	Italy	Pisa	Strain in EMJH medium	Hedgehog	75.8	Leptospira	known
Leptospira _ Sejroe _M84	Denmark	NK	Strain in EMJH medium	House mouse (*Mus musculus spicilegus*)	75.6	Leptospira	known
Leptospira _ Tarassovi _ Mitis Johnson	Australia	NK	Strain in EMJH medium	Man	75.8	Leptospira	known
Leptospira serovar Ballum strain Mus 127	Denmark	NK	Strain in EMJH medium	Mouse (*Mus musculus*)	75.8	Leptospira	known
Leptospira serovar Saxkoebing	Denmark	NK	Strain in EMJH medium	Yellow necked field	75.6	Leptospira	known

strain Mus 24				mouse (*Apodemus flavicollis*)			
Leptospira serovar Hardjo strain Harjoprajitno	Indonesia	Sumatra	Strain in EMJH medium	Man	75.8	Leptospira	known
Brucella B1 625 -2019	Botswana	Lobatse	SC-DNA	Bovine	83.2	Brucella	known
Brucella B2 646 - 2019	Botswana	Lobatse	Culture-DNA	Bovine	83.8	Brucella	known
Brucella B3 050 - 2019	Botswana	Lobatse	Lung culture -DNA	Bovine	83	Brucella	known
Brucella B4 652 -2019	Botswana	Lobatse	SC-DNA	Bovine	82.8	Brucella	known
Bot 2332/2014	Botswana	Lobatse	SC	Caprine	NA	Negative	Blind
Bot 1024/2016	Botswana	Ramotswa	Tissues	Caprine	NA	Negative	Blind
Bot 2929/2017	Botswana	Mahalapye	Tissues	Caprine	NA	Negative	Blind
Bot 2947/2017	Botswana	Gaborone	Tissues	Caprine	NA	Negative	Blind
Bot 2992/2017	Botswana	Gaborone	Tissues	Caprine	NA	Negative	Blind
Bot 3223/2017	Botswana	Gaborone	Tissues	Caprine	NA	Negative	Blind
Bot 3224/2017	Botswana	Kanye	Tissues	Caprine	NA	Negative	Blind
Bot 3177/2017	Botswana	Ramotswa	Tissues	Caprine	NA	Negative	Blind
Bot 3531/2017	Botswana	Ramotswa	Placenta	Caprine	NA	Negative	Blind
Bot 1100/2019	Botswana	Kasane	Tissues	Ovine	NA	Negative	Blind
Bot 3040/2019	Botswana	Molepolole	SC/Tissues	Caprine	NA	Negative	Blind
Bot 2825/2020	Botswana	Lobatse	Milk	Bovine	NA	Negative	Blind
Bot 1633/2020	Botswana	Gaborone	Milk	Bovine	NA	Negative	Blind
Bot 1760/2020	Botswana	Lobatse	Milk	Bovine	NA	Negative	Blind
Bot 2654/2020	Botswana	Palapye	Milk	Bovine	NA	Negative	Blind
Bot 2822/2020	Botswana	Lobatse	Milk	Bovine	NA	Negative	Blind
Bot 1608/2020	Botswana	Lobatse	Milk	Bovine	NA	Negative	Blind
Bot 1606/2020	Botswana	Lobatse	Milk	Bovine	NA	Negative	Blind
Bot 1763/2020	Botswana	Lobatse	Milk	Bovine	NA	Negative	Blind
Bot 1630/2020	Botswana	Gaborone	Milk	Bovine	NA	Negative	Blind
Bot 1644/2020	Botswana	Ramotswa	FT	Caprine	NA	Negative	Blind
Bot 1469/2020	Botswana	Lobatse	Milk	Bovine	NA	Negative	Blind

Bot 2094/2020	Botswana	Gaborone	Milk	Bovine	NA	Negative	Blind
Bot 2097/2020	Botswana	Lobatse	Milk	Bovine	NA	Negative	Blind
Bot 1986/2020	Botswana	Gaborone	Milk	Bovine	NA	Negative	Blind
Bot 1766/2020	Botswana	Gaborone	Milk	Bovine	NA	Negative	Blind
Bot 3183/2020	Botswana	Lobatse	Milk	Bovine	NA	Negative	Blind
Bot 3351/2020	Botswana	Lobatse	Milk	Bovine	NA	Negative	Blind
Bot 1570/2020	Botswana	Gaborone	Milk	Bovine	NA	Negative	Blind
Bot 2175/2020	Botswana	Mochudi	SC/FT	Caprine	NA	Negative	Blind
Bot 1792/2020	Botswana	Mochudi	Tissues	Caprine	NA	Negative	Blind
Bot 3671/2020	Botswana	Lobatse	Serum	Bovine	NA	Negative	Blind
Bot 3676/2020	Botswana	Lobatse	Serum	Bovine	NA	Negative	Blind
Bot 1802/2020	Botswana	Ramotswa	Tissues	Caprine	NA	Negative	Blind
Bot 2116/2020	Botswana	Maun	Tissues	Caprine	NA	Negative	Blind
Bot 2419/2020	Botswana	Nata	FT	Caprine	NA	Negative	Blind
Bot 2428/2020	Botswana	Molepolole	FT	Caprine	NA	Negative	Blind
Bot 902/2021	Botswana	Molepolole	FT	Caprine	NA	Negative	Blind
Bot 0439/2021	Botswana	Palapye	WB	Caprine	NA	Negative	Blind
Bot 0735/2021	Botswana	Shakawe	Tissues	Caprine	NA	Negative	Blind
Bot 0711/2021	Botswana	Lobatse	Serum	Bovine	NA	Negative	Blind
Bot 0677/2021	Botswana	Kanye	Serum	Bovine	NA	Negative	Blind
Brucella abortus 1	South Africa	NK	Bacterial culture	Bovine	83.0	Brucella	known
Brucella abortus 2	South Africa	NK	Bacterial culture	Bovine	83.0	Brucella	known
Brucella abortus 3	South Africa	NK	Bacterial culture	Bovine	83.0	Brucella	known
Brucella abortus 7	South Africa	NK	Bacterial culture	Bovine	83.0	Brucella	known
Brucella abortus 8	South Africa	NK	Bacterial culture	Bovine	83.0	Brucella	known
Brucella abortus 9	South Africa	NK	Bacterial culture	Bovine	83.2	Brucella	known
Brucella abortus 10	South Africa	NK	Bacterial culture	Bovine	83.2	Brucella	known
Brucella abortus 11	South Africa	NK	Bacterial culture	Bovine	83.0	Brucella	known
Brucella abortus 12	South Africa	NK	Bacterial culture	Bovine	83.0	Brucella	known
Brucella abortus 13	South Africa	NK	Bacterial culture	Bovine	83.0	Brucella	known
Brucella abortus 14	South Africa	NK	Bacterial culture	Bovine	83.0	Brucella	known
Brucella abortus 15	South Africa	NK	Bacterial culture	Buffalo	83.0	Brucella	known
Brucella abortus 16	South Africa	NK	Bacterial culture	Bovine	83.0	Brucella	known
Brucella abortus 17	South Africa	NK	Bacterial culture	Bovine	83.0	Brucella	known

Brucella abortus 18	South Africa	NK	Bacterial culture	Bovine	83.0	Brucella	known
Brucella abortus 19	South Africa	NK	Bacterial culture	Bovine	83.2	Brucella	known
Brucella abortus 20	South Africa	NK	Bacterial culture	Bovine	83.2	Brucella	known
Brucella melitensis 2	South Africa	NK	Bacterial culture	Goat	83.2	Brucella	known
Brucella melitensis 3	South Africa	NK	Bacterial culture	Goat	83.2	Brucella	known
Brucella ovis 1	South Africa	NK	Bacterial culture	Sheep	83.2	Brucella	known
Brucella ovis 2	South Africa	NK	Bacterial culture	Sheep	83.2	Brucella	known
Brucella ovis 3	South Africa	NK	Bacterial culture	Sheep	83.2	Brucella	known
Brucella suis 2	South Africa	NK	Bacterial culture	Pig	83.2	Brucella	known
Brucella suis 3	South Africa	NK	Bacterial culture	Pig	83.2	Brucella	known
Brucella abortus bv9, NCTC 10507	UK	NK	Bacterial culture	NK	83.2	Brucella	known
Brucella melitensis 16m, NCTC 10094	UK	NK	Bacterial culture	NK	83.2	Brucella	known
Brucella ovis 63/290, NCTC 10512	UK	NK	Bacterial culture	NK	83.2	Brucella	known
Brucella suis bv3 686, NCTC 10511	UK	NK	Bacterial culture	NK	83.0	Brucella	known
Coxiella burnetii	South Africa	NK	Tissue	Cattle	80.8	Coxiella	known
*Coxiella burnetii*a 2	South Africa	NK	Tissue	Cattle	80.8	Coxiella	known
Coxiella burnetii 3	South Africa	NK	Tissue	Cattle	80.8	Coxiella	known
Coxiella burnetii 4	South Africa	NK	Tissue	Cattle	80.8	Coxiella	known
Coxiella burnetii 5	South Africa	NK	Tissue	Cattle	80.8	Coxiella	known
Coxiella burnetii 6	South Africa	NK	Tissue	Cattle	80.8	Coxiella	known
Coxiella burnetii 7	South Africa	NK	Tissue	Cattle	80.8	Coxiella	known
Coxiella burnetii 8	South Africa	NK	Vaginal swab	Sheep	80.8	Coxiella	known
Coxiella burnetii 10	South Africa	NK	Vaginal swab	Sheep	80.8	Coxiella	known
Coxiella burnetii 11	South Africa	NK	Vaginal swab	Sheep	80.8	Coxiella	known
Coxiella burnetii 12	South Africa	NK	Vaginal swab	Sheep	80.8	Coxiella	known
Coxiella burnetii 13	South Africa	NK	Tissue	Goat	80.6	Coxiella	known
Coxiella burnetii	South	NK	Tissue	Goat	80.8	Coxiella	known

14	Africa						
Coxiella burnetii 15	South Africa	NK	Tissue	Goat	80.6	Coxiella	known
Coxiella burnetii 17	South Africa	NK	Tissue	Goat	80.8	Coxiella	known
Leptospira Canicola-Grippotyphosa-Hardjo-Icterohaemorrhagiae-Pomona Bacterin 1	South Africa	NK	Leptoferm-5 vaccine (Zoetis)	NA	75.8	Leptospira	known
Listeria monocytogenes 1	South Africa	NK	Bacterial culture	NK	77.6	Listeria	known
Listeria monocytogenes 3	South Africa	NK	Bacterial culture	NK	77.4	Listeria	known
Listeria monocytogenes 5	South Africa	NK	Bacterial culture	NK	77.6	Listeria	known
Listeria monocytogenes 9	South Africa	NK	Bacterial culture	NK	77.6	Listeria	known
Listeria monocytogenes 10	South Africa	NK	Bacterial culture	NK	77.6	Listeria	known
Listeria monocytogenes 11	South Africa	NK	Bacterial culture	NK	77.6	Listeria	known
Listeria monocytogenes 13	South Africa	NK	Bacterial culture	NK	77.6	Listeria	known
Listeria monocytogenes 14	South Africa	NK	Bacterial culture	NK	77.6	Listeria	known
Listeria monocytogenes 15	South Africa	NK	Bacterial culture	NK	77.6	Listeria	known
Listeria monocytogenes 16	South Africa	NK	Bacterial culture	NK	77.6	Listeria	known
Listeria monocytogenes 17	South Africa	NK	Bacterial culture	NK	77.6	Listeria	known
Listeria monocytogenes 18	South Africa	NK	Bacterial culture	NK	77.6	Listeria	known
Listeria monocytogenes 19	South Africa	NK	Bacterial culture	NK	77.6	Listeria	known
Listeria monocytogenes 20	South Africa	NK	Bacterial culture	NK	77.6	Listeria	known

Abbreviations: AF = Abdominal fluid; FT = Foetal tissues; NK = Not known; SC = Stomach contents; WB = Whole blood

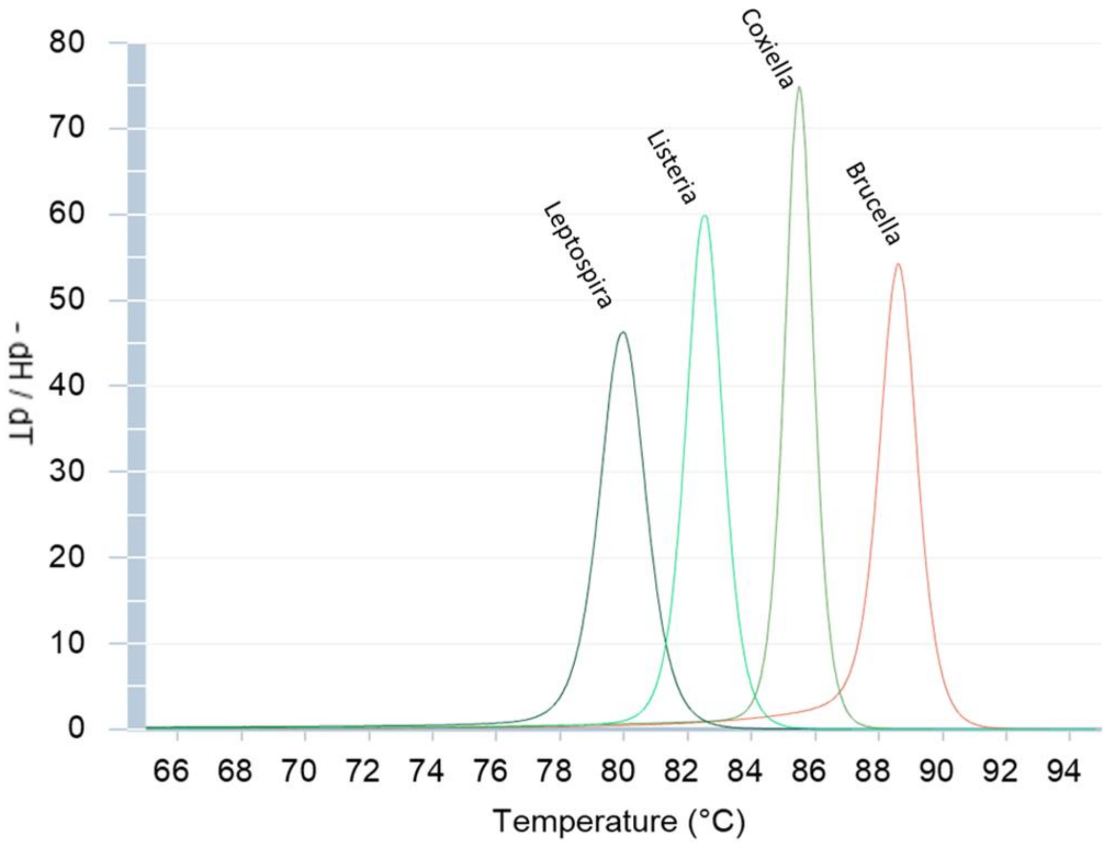

Figure S1: Umelt simulation graph showing melting curve, the negative derivative of the fluorescence (F) over temperature (T) (dF/dT) against the temperature (T).

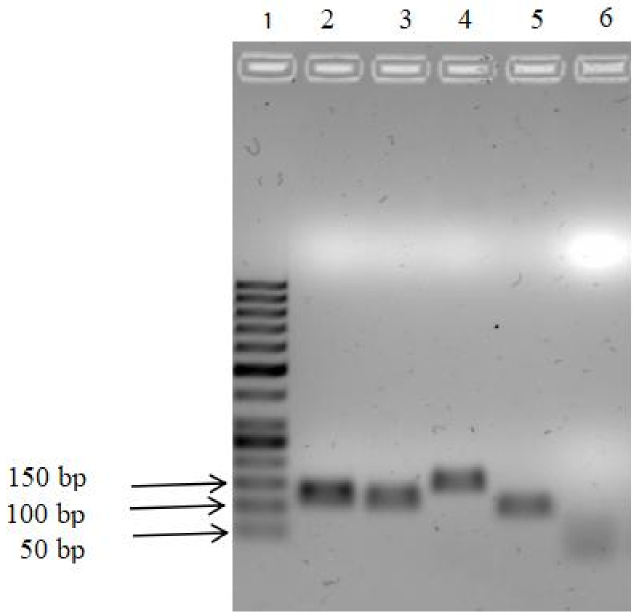

Figure S2: Gel picture showing the expected amplicon size of each of the primers. Lane 1: 50-bp ladder; lane 2: *Brucella* spp. (101 bp) ; lane 3: *Listeria monocytogenes* (93 bp); lane 4: *Coxiella burnetii* (121); lane 5: *Leptospira* spp (77 bp); lane 6: Negative control without DNA.
